# Dr. Huggan, on the Vaccine Inoculation

**Published:** 1800-03

**Authors:** 


### *To the Editors of the Medical and Physical Journal.*

GENTLEMEN, *Plymouth*, *Dec.* 31, 1799.

THE introduction of the Cow-pox into practice, as a substitute for the Small-pox, having been found to be expedient, in the most extensive sense of the word, the discussion of the subject will, of course, be considered as closed. This is a circumstance truly honourable to Dr. JENNER, by whom this beneficial improvement, doubtless one of the most important in Medicine, has been first made known to the world.

> Exegit monumentum, ære perennius,

> Quod non ——

> Possit diruere, aut innumerabilis

> Annorum, series, et fuga temporum.

The zeal with which Dr. Pearfon did, as is well known, fo early and affiduoufly inveftigate all the facts belonging to this new difeafe; and, when fatisfied, from his own experience, of the propriety of introducing it into practice, his further endeavours to make his fentiments as publicly known, and the advantages of the *new inoculation* as extenfively felt by the community at large as poffible, do equal honour to his feelings as a man, and his judgement as a phyfician. Their opinion of its utility, and that of Dr. Woodville, (defervedly carrying much weight from his high profeffional rank) having been confirmed by the reports of fome of the moft refpectable of the profeffion, not only in Britain, but on the continent; there is not much fear now of the public confidence being likely to be weakened by the mifreprefentations of perfons interefted in oppofing its progrefs, far lefs from the "ignorance" and "prefumption of fciolifts."

Notwithftanding, however, and what will hardly be credited, the introduction of the new practice here, as well as in fome other parts of this county, has been oppofed by profeffional men with a degree of acrimony very unbecoming the medical character.—"Reptiles that plant themfelves in the high road of improvement, and try to hifs back all that would advance."

Failure in inoculation, independent of prejudice, has contributed in a great meafure to retard the progrefs of the new inoculation here, till of late, Mr. Stewart, an active and intelligent furgeon in Plymouth, has been more fortunate in that refpect. The mildnefs with which all his patients had the difeafe (aftonifhing in the eyes of people who had been told fo many ridiculous ftories about its beftial nature,) has, on the recommendation of Dr. Remmett, the principal phyfician here, induced fome of the refpectable inhabitants to have the difeafe introduced into their families. From the judicious arrangements which Mr. Stewart had made in his inoculations, he has, hitherto, been able to procure the matter in a recent ftate from the arm of a patient, when he wanted it for that purpofe, (as that has been enjoined as a circumftance requiring particular attention,) and is in hopes of being able to do the fame through the winter; fo that the benefits of the new inoculation will foon be as readily acknowledged, and generally experienced, in this town and neighbourhood, as in other parts of the kingdom. When I hear of medical men ridiculing the cow-pox, feemingly with the view of preventing its advantages from being experimentally afcertained by that part of the community, who have been wheedled into a miftaken confidence in their candour, I cannot help fufpecting the oppofition of fuch men to proceed from motives, other than thofe of

*of conviction*, eſpecially if their profeſſional rank does not allow us to impute to them *ignorance*, or *want* of *diſcernment*. What medical man of candour, or profeſſional information, would attempt to reſiſt the force of ſo great and incontrovertible a maſs of evidence of the utility of the new practice, or the high authority on which it has been ſo ſtrongly recommended?

Having allowed myſelf to doubt the expediency of this new inoculation, from the peruſal of an anonymous letter in the Annals of Medicine (Vol. III. p. 446,) when I was in Ireland, I was not undeceived till ſome time after my return to England, when I met with Vol. II. of the Medicina Nautica, in which the benevolent and learned author had inſerted Dr. Pearſon's Corollaries on the Cow-pox, ſeemingly with the view of recommending the introduction of it into the navy, in addition to the other improvements that have been made in that important department of the public ſervice, ſince his appointment of Phyſician of the Fleet. Induced by ſo great an authority, I readily embraced the firſt opportunity of trying the new practice that ſhould occur; and though my firſt experiment, which was made in September laſt, was not very encouraging, yet I was determined to perſevere, till I could, from my own experience, form a decided opinion on the ſubject, which, I think, I have been able to do to my ſatisfaction; and from the reſult of which ſhall, with your permiſſion, take the liberty of briefly ſtating the ſuperior advantages of the Vaccine Inoculation over the Variolous. The firſt caſe was from particular, though unavoidable circumſtances, rather an exception to our after-experience of the mildneſs of the diſeaſe.

A woman, (the only adult whom I have inoculated,) had the eruptive fever, equally as ſevere as it is commonly in the ſmall-pox with grown people, the inflammation of the arm more troubleſome, but, upon the whole, the period of the diſeaſe was ſhorter, in conſequence of the abſence of eruptions.

The eruptive fever was milder than in the ſmall-pox; in infants, barely perceptible.

During the variolous eruptive fever in children, the eruption is ſometimes uſhered in with epileptic fits, which, though generally the fore-runners of a mild diſeaſe, are nevertheleſs alarming, and have, in ſome inſtances, proved fatal. Theſe did not accompany the eruptive fever in any of our patients. In one caſe of a female child two years old, the mother was alarmed from her ſtarting ſuddenly, drawing in her arms and legs, and writhing her body, as if in pain, as was ſuppoſed, from her crying ſo vehemently at the time.

A ſore throat, or painful and tedious abſceſſes about the axilla; illa, often accompany, or immediately follow the ſmall-pox. None of my patients (except the woman before mentioned) had a ſingle troubleſome ſymptom after the fever had gone off; nor are any of them likely to be afflicted with ſcrofula, &c. which have been known to follow the ſmall-pox, even under its mildeſt form.

In the ſmall-pox often, and that after every appearance of danger has ſubſided, the diſeaſe is rendered tedious from the number of the puſtules, &c. The trifling eruption which a few of my patients had, did not in the ſmalleſt degree protract the diſeaſe.

In the ſmall-pox, eſpecially if the eruptive fever is violent, and the eruptions numerous and painful, emetics, opiates, &c. are indiſpenſably neceſſary. Only one of my patients required any medicine.

Another advantage of the cow-pox, as we learn from Dr. Jenner, but which, in the preſent ſtate of its progreſs, muſt not yet be counted upon, is, that it is never communicated by effluvia; therefore, it would appear from this, that, however ſimilar in their appearances, &c. the variolous and vaccine are, they are really different ſpecific diſeaſes.* Now if, after a general inoculation, (a circumſtance which, from the Reverend Mr. Holt's letter, appears to be neither impracticable nor likely to be attended with much public inconvenience) every child was inoculated, at a certain age, with cow-pox matter, we might reaſonably calculate that the period was not far diſtant, when one of the moſt loathſome diſeaſes, and moſt deſtructive of human comfort, of any to which the human race is liable, would be entirely extinguiſhed, and when it would become equally as unknown in this iſland, although not leſs dreaded, than the plague is by us of the preſent day.

As it ſeems at length to be agreed on all hands, that the human body, after having undergone the vaccine, is perfectly ſecure againſt the infection of the variolous diſeaſe, it is certainly but of ſecondary conſideration, whether the latter renders the body

* Some of the Readers of this Journal, who are of the ſame opinion, may, perhaps, conſider the confidence with which the contrary has been ſo roundly affirmed, (*Monthly Magazine for June laſt*) as bordering upon preſumption. It has been aſſerted, and that without the leaſt ſhadow of proof, that the Cow-pox is only the Small-pox having undergone a certain modification, by paſſing through the quadruped; and that it will degenerate into Small-pox again, by paſſing a ſeries of times from one human being to another, without interruption. The author of this opinion, however, not ſatiſfied with the grounds on which he reſts it, intends, he tells us, to determine this point by experiments, the reſult of which he promiſes to lay before the public.

body unfufceptible of the former, as this is, when compared with the confluent fmall-pox a very mild difeafe; and as it is not received by effluvia, it would feem to be in fome degree voluntary, on the part of any individual, to have it at all.—But local puftules only, are by no means allowed to be a proof of any perfons having had either the one or the other of thefe difeafes.

T. R. aged feventeen years, was in June 1795, inoculated for the fmall-pox; a puftule containing matter, appeared upon the punctured part, but without any conftitutional fever. In the fpring 1797, he was taken ill with fmall-pox, which proved of the confluent kind, of which, after many fears entertained about the event of his recovery, he at length got well. Whilft writing this, I have upon my left arm a vaccine puftule from inoculation that has been very painful, without, however, any other confequences.

[ To be continued in our next Number. ]

